# Traditional Chinese Medicine Alleviates Ulcerative Colitis via Modulating Gut Microbiota

**DOI:** 10.1155/2022/8075344

**Published:** 2022-03-09

**Authors:** Wan Feng, Lei Zhu, Hong Shen

**Affiliations:** Department of Gastroenterology, Affiliated Hospital of Nanjing University of Chinese Medicine, Nanjing, China

## Abstract

Ulcerative colitis (UC) is a chronic inflammatory bowel disorder characterized by relapsing and remitting inflammation of the bowel. In recent decades, traditional Chinese medicine (TCM) has been widely used in the therapy of UC. However, its underlying mechanisms have not been sufficiently elucidated. Accumulating studies indicate that the gut microbial dysbiosis is closely related to UC. It has been demonstrated that TCM could alter the composition of intestinal microbiota by enriching beneficial and SCFA-producing bacteria and reducing pathogenic bacteria. In this review, we discussed recent evidence regarding the TCM and its role in modulating gut microbiota for the treatment of UC.

## 1. Introduction

Ulcerative colitis (UC) is an idiopathic inflammatory disorder affecting the large intestine. It is generally characterized by relapsing and remitting inflammation of the bowel. The main clinical symptoms include abdominal pain, diarrhea, and bloody stool [1]. An estimated 1 million individuals in the United States and 2.5 million people in Europe suffer from inflammatory bowel disease (IBD) [2]. The incidence of UC is increasing rapidly in Asia. Data from a systematic review of epidemiological studies indicated the incidence rates for IBD and UC were 1.80 and 1.33/1,000,000 in mainland China [3, 4].

The exact etiology and pathogenesis of UC remain unclear. Multiple factors contributed to the development of UC, such as genetic predisposition, immunological dysregulation, environmental exposure, and disorder of intestinal microbiota [5]. The current therapy includes 5-aminosalicylic acid drugs, steroids, immunosuppressants, biologic agents, and complementary and alternative medications. In recent decades, traditional Chinese medicine (TCM) has increasingly been used as an alternative treatment [6, 7]. In this review, we present recent evidence regarding the TCM and its role in modulating gut microbiota for the therapy of UC.

## 2. Traditional Chinese Medicine and UC

Ulcerative colitis (UC) is a chronic, relapsing disease requiring lifelong treatment. Effective and tolerable medical choice is urgently needed. As a major form of complementary and alternative medicine, Chinese herbal medicine has been commonly practiced in Asia.

Traditional Chinese medicine (TCM) is widely accepted by patients with chronic disease due to its notable efficacy and natural property. The adverse effects of TCM should not be ignored. The clinical study evaluating the efficacy of TCM among patients with UC suggests that TCM do not appear to have significant adverse events compared to control groups [8]. However, some studies observed liver dysfunction, nausea, and pulmonary arterial hypertension among UC patients who received TCM [9, 10]. Although safe and well tolerated, doctors should recognize its potential side effects [11].

Growing evidence indicated that many herbal medicines, such as *Aloe vera* gel, indigo naturalis, and *Andrographis paniculata* extract, could improve clinical symptoms, reduce the recurrence rate, and increase the response rate [12–14]. In a multicenter, double-blind trial, patients were randomly assigned to indigo naturalis (IN) or placebo for 8 weeks. Clinical remission was observed in 55.0% patients on IN compared to 4.5% on placebo [9].

Fufangkushen colon-coated capsule (FCC) is a Chinese herbal drug used for the management of UC. A randomised double-blind study assessed the therapeutic potential of FCC in patients of active UC. 320 active UC patients were assigned to FCC or mesalazine for 8 weeks. At week 8, 72.5% patients treated with FCC and 65% patients on mesalazine obtained a clinical response [15]. In another double-blind clinical trial, ulcerative proctitis patients were randomised to Xilei-san enema or dexamethasone enema. They found no significant difference in terms of clinical and endoscopic remission between the Xilei-san enema and the conventional steroid enema group [16].

Qingchang Huashi (QCHS) is a Chinese herbal formula used for UC patients by the Affiliated Hospital of Nanjing University of Chinese Medicine. Our study evaluating the clinical effects of QCHS showed encouraging results. 60 UC patients were randomised to QCHS or mesalazine for 8 weeks. An improvement in endoscopic score was found in the two groups. There was no significant difference in terms of mucosal healing and the clinical remission between the QCHS and mesalazine groups [17]. It was also demonstrated in another randomised double-blind multicenter study. 119 patients were randomised to QCHS granules or an identical placebo with continued 5-ASA therapy. 31.48% patients on the QCHS group and 12.50% patients on placebo obtained a clinical remission. 92.59% and 72.92% patients on QCHS and placebo groups achieved a clinical response [10].

## 3. Gut Microbiota and UC

Numerous studies suggest that alteration of the intestinal microbiota is intimately related to some chronic disease, including heart failure, diabetes mellitus, irritable bowel syndrome, and IBD [18–23].

Growing studies suggested that the diversity and metabolic activity of the gut flora in UC is altered compared with that in healthy adults [24, 25]. In UC patients, a reduction in the abundance of *Firmicutes, Clostridium* cluster, and an increased level of *Proteobacteria* have been found [26–29]. A systematic review evaluated the relationship between gut microbiota, and IBD found *Eubacterium rectale* and *Akkermansia* were reduced; whereas, the level of *E. coli* is enriched in patients with UC. Furthermore, at the phylum level, a loss in phyla Tenericutes and Lentisphaerae was observed in UC patients [30].

Remodeling the gut flora emerges as an attractive approach for clinical therapy for patients with UC, including probiotics and fecal microbiota transplantation (FMT) [26, 31]. A randomised, double dummy study evaluating the efficacy of probiotics showed promising results. When compared with mesalazine, *Escherichia coli* Nissle 1917 showed similar efficacy in maintaining remission [32, 33]. Restoring the dysbiosis with FMT has been discussed in some clinical studies [34]. A meta-analysis of 4 randomised controlled trials evaluated the efficacy of FMT in inducing disease remission. Clinical remission was found in 39 of 140 UC patients on FMT compared to 13 of 137 patients on placebo [35].

## 4. Mechanisms of TCM Alleviating UC via the Gut Microbiota

### 4.1. Gut Microbiota Mediated Biotransformation of TCM

Herb medicines exhibit poor absorption and bioavailability. When consumed orally, TCMs reach the gut and interact with intestinal microbiota. For example, many TCMs contain polyphenols, but the polyphenols show less than 10% absorption and about 90–95% of the polyphenols accumulate in colon [36]. Growing evidence demonstrates orally administrated TCM is affected by enzymes of gut microbiota [37, 38]. Nitroreductase and azoreductase are two major reductases in gut bacteria. Feng et al. reported that the intestinal bacteria could convert berberine into absorbable form of dihydroberberine (dhBBR) [39].

Studies have also confirmed that gut microbiota could transform TCM by various reactions, such as oxidation, reduction, isomerization, and hydrolysis [40, 41]. For example, baicalin is often used for treatment of UC. It has been reported that intestinal bacteria produced beta-glucuronidase, which could hydrolyze baicalin into baicalein [42, 43].

### 4.2. Regulation of Gut Microbiota Composition

Accumulating evidence indicated herb medicine could modulate the composition of intestinal microbiota [43–47]. TCM could promote the growth of beneficial bacteria and reduce the abundance of some pathogenic bacteria. Emerging evidence indicates that many herbal medicines, such as Red Ginseng, Semen Coicis, Kuijieling, and triptolide, hold promise for the management of colitis via improving gut microbiota [48–52].

Huangqin decoction (HQD) is a classical Chinese medicine formula clinically used to treat colitis for centuries. Yang et al. reported HQD attenuated colitis by restoring microbiota dysbiosis. The relative abundance of *Lactococcus* was enhanced; whereas, *Desulfovibrio* and *Helicobacter* were reduced in the HQD group [53]. Baitouweng (BTW) decoction has been applied to attenuate the symptoms of UC. Gut microbial populations significantly changed after BTW decoction administration. For instance, the ratio of *Firmicutes* to *Bacteroidetes* and the abundance of *Proteobacteria* was reduced. At the genus level, the amount of *Escherichia-Shigella* was inhibited; whereas, *Lactobacillus* and *Akkermansia* were enriched [54].

Lizhong decoction (LZD), a Chinese medicine formula, has been utilized in the therapy of gastrointestinal dysfunction. LZD attenuates inflammation in animal models of colitis by inhibiting the amounts of harmful bacteria such as *Clostridium sensu stricto*, *Enterobacter*, and *Escherichia-Shigella* and increasing the proportion of beneficial bacteria [55].

### 4.3. Regulation of Microbiota Metabolites

Gut microbiota metabolites, such as short-chain fatty acids (SCFAs) and bile acids (BAs) closely affect the development of IBD. Multiple studies showed lower relative abundance of SCFA-producing bacteria, for instance, *Faecalibacterium*, *Clostridium* clusters IV and XIVb, *Roseburia*, *Odoribacter*, and *Leuconostocaceae* in UC patients [56, 57]. TCM has been found to adjust the gut flora structure and metabolic profiles [37, 58, 59]. It is reported that SCFAs exert their effects through activating G-protein-coupled receptors (GPCRs) and inhibiting histone deacetylases (HDACs) [60–62].

Recent studies showed that many TCMs could enrich the abundance of SCFA-producing bacteria in models of UC. A recent animal study found that indigo naturalis could ameliorate intestinal dysbiosis. It elevated the level of fecal butyrate and the abundance of *Ruminococcus_1* and *Butyricicoccus*. Furthermore, an increased level of short chain fatty acid-associated receptors GPR41 and GPR43 was found [63].

Shenling Baizhu San (SLBZS) exhibits efficacy in treating UC for centuries [64]. SLBZS administration enhanced the amount of SCFA-producing bacteria, such as *Prevotella* and *Oscillospira*, and decreased pathogenic bacteria including *Desulfovibrio* and *Bilophila* [65]. Baicalin is an active component derived from Huangqin, a traditional herb medicine extensively used for UC. Our study found that baicalin increased the amount of butyrate-producing bacteria such as *Butyricimonas* spp., *Roseburia* spp., and *Eubacteria* spp. and level of fecal butyrate [66].

Bile acids (BAs) are produced from cholesterol and metabolized by the intestinal flora. BAs exert its effects by activating the nuclear BA receptor farnesoid X receptor (FXR) and G protein-coupled BA receptor 1 (TGR5) [67, 68]. Emerging evidence suggests that dysmetabolism of BA is intimately linked to the development of UC. Hu et al. indicated that Qingchang Huashi (QCHS) formula could alter the gut flora structure and regulate the bile acid metabolism [69]. Recent study showed Baitouweng (BTW) decoction administration promoted the expression of TGR5 and FXR. The authors also suggest that the therapeutic potential of BTW decoction is correlated with modulation of the gut microbiota and bile acids [70].

### 4.4. Regulation of Th17/Treg Balance

Growing studies indicate that gut microbiota play a vital role in the immune homeostasis, especially in the Th17/Treg balance [71, 72]. Some specific bacteria have been involved in the Treg development and T cell responses, such as segmented filamentous, *Lactobacillus* and *Bifidobacterium* [73]. Gut microbiota and its metabolites have been found to regulate immune homeostasis. SCFAs are reported to be associated with the regulation of colonic regulatory T cells [59, 60].

A recent animal study found berberine (BBR) could reduce the relative abundance of *Desulfovibrio* and enrich *Eubacterium* strains. Additionally, BBR could modulate the Treg/Th17 balance through altering gut flora. It is reported that BBR administration does not affect Treg/Th17 after depletion of gut flora [74]. Zhang et al. found *Abelmoschus manihot* alleviated colitis in mice via adjusting the structure of gut microbiota and Th17/Treg balance. It also increased the microbial metabolites in feces especially butyrate and acetate [75].

Rhubarb Peony decoction (RPD) is commonly used in the treatment of UC for centuries. RPD promoted the growth of *Butyricicoccus pullicaecorum, Firmicutes*, and *Actinobacteria* and reduced amount of *Proteobacteria* and *Bacteroidetes*. Recent study demonstrates that RPD alleviates colitis by modulating gut flora to restore Th17/Treg homeostasis. It also reduced the level of proinflammatory cytokines, such as IL-6 and TNF-*α*, and enhanced the production of Treg-related cytokine TGF-*β* [76].

### 4.5. Regulation of Intestinal Barrier

Normal gut bacteria play a role in protecting intestinal barrier. Alterations and disruptions to the intestinal barrier are observed in patients with UC [77]. Several studies have reported gut microbiota and its metabolites altered intestinal permeability and promoted the level of intestinal tight junction proteins, such as claudin, and zonula occludens 1 (ZO-1) [78–80]. It has been reported that TCM could protect intestinal barrier via microbiota.

Qingchang Wenzhong decoction (QCWZD), a classical Chinese herbal formula, exerts beneficial effects on UC. Sun et al. reported enhanced expression levels of intestinal tight junction proteins and increased numbers of goblet cells in QCWZD-treated mice. They also observed increased amount of *Lactobacillus* and inhibited pathogenic species, such as *Bacteroides* and *Streptococcus*. To further confirm that QCWZD could improve the intestinal barrier by altering the gut microbiota, they applied fecal microbiota transplantation and found fecal transplantation from QCWZD-treated mice could accelerate intestinal epithelial wound healing [81].

The total flavone of *Abelmoschus manihot* (TFA), the major pharmacological component of Flos *Abelmoschus manihot*, has been used in the alleviation of UC. Wang et al. found that TFA promoted the level of MUC2, KLF4, and ZO-1 proteins and ameliorated gut microbial dysbiosis. Furthermore, TFA fecal microbiota transplantation alleviated intestinal barrier impair in UC mice [82].

## 5. Conclusion and Perspectives

Growing studies suggest gut microbiota is a new target for TCM in treating UC. For example, TCM could enrich beneficial bacterial species and decrease harmful bacteria during UC treatment. To better elucidate the mechanism of TCM in alleviating UC, basic research and clinical investigation are expected to explore the specific bacterial strains involved in the metabolism of TCM. Most studies assessing the association of gut flora and TCM are based on mice or animal models. However, such studies in humans are lacking.

Syndrome differentiation is one of the important TCM concepts. Syndrome differentiation refers to comprehensive analysis of clinical information. For example, UC can be divided into Pi-Xu-Shi-Yun syndrome and Da-Chang-Shi-Re syndrome. Zhang et al. found that the gut microbiota is different between the two TCM syndromes of UC [83]. Future studies on the syndrome-based microbiota may improve the personalized TCM interventions [84].

Due to complexity and personalized properties of TCM, scientific explanation for the efficacy and underlying mechanisms of TCM is limited. Specific molecules and bioactive ingredients purified or isolated from herb medicines provide new insight into the understanding and acceptance of TCM [59]. Additionally, enzymes encoded by intestinal bacteria and microbe-derived metabolites could be the biomarkers for the intervention of TCM [44]. Further study using integrative approaches, such as metagenomics, metaproteomics, metatranscriptomics, and metabolomics, is needed to reveal potential biomarkers.

## Data Availability

The data generated or analyzed during this study are included within this article.
